# Development and evaluation of an elective course on the pharmacist’s role in disaster management in France

**DOI:** 10.3352/jeehp.2019.16.19

**Published:** 2019-07-15

**Authors:** Marc Montana, Fanny Mathias, Pascal Rathelot, Jérôme Lacroix, Patrice Vanelle

**Affiliations:** 1 Aix Marseille Université, CNRS, Institut de Chimie Radicalaire, Marseille, France; 2 Assistance Publique-Hôpitaux de Marseille, Oncopharma, Marseille, France; 3 Military Health Service (SSA), Joint Medical Supply Depot (ERSA), Marseille, France; 4 Assistance Publique-Hôpitaux de Marseille, Central Service for Pharmaceutical Quality and Information (SCQIP), Marseille, France; Hallym University, Korea

**Keywords:** Crisis resource management, Pharmacy, Pharmacy students, Terrorism, France

## Abstract

**Purpose:**

To describe our experiences with the development of an elective course on the pharmacist’s role in disaster management for third-year pharmacy students and to evaluate its effects on students’ knowledge and their perceptions of the introduction of this course into the curriculum.

**Methods:**

An expert team of physicians, surgeons, and pharmacists of the Service de Santé des Armées, pharmacists teaching at the Faculty of Pharmacy, and pharmacists from the Bataillon des Marins Pompiers de Marseille developed a program consisting of 30 hours of modules on disaster response training based on previously published recommendations, a literature analysis, and international guidelines. Students’ knowledge of key competencies was assessed after some teaching sessions through a multiple-choice quiz. Students’ self-perceived knowledge, perceptions of teaching quality, and degree of satisfaction were evaluated through a voluntary survey after the last teaching session on November 15.

**Results:**

The final curriculum consisted of 6 modules. Students’ knowledge of key competencies was assessed using multiple-choice quizzes, with a mean score of 19 of 25. Almost all students (98.3%) reported that this training program improved their knowledge of the pharmacist’s role in disaster management, and 79.3% stated that they would recommend this optional course.

**Conclusion:**

This training course demonstrated the potential to increase the number of pharmacists prepared to respond to disasters. It also expanded students’ understanding of the pharmacist’s role and stimulated their interest in emergency preparedness. Further refinement of the program, including a simulation of mass triage in an emergency setting, will be conducted next year.

## Introduction

Recent terrorist attacks, coupled with a high number of natural disasters, have had a destabilizing impact on the general population, causing distress and fear and clearly indicating the need to include disaster preparedness training as part of the curriculum of all educational programs for the health professions [1]. Although medical schools in the United States are encouraged to follow the recommendations of the Association of American Medical Colleges to develop a disaster medicine module for inclusion in the curriculum of medical students, few medical schools explicitly include practical education on disasters and health threats in their curriculum [2]. In addition, 91.4% of medical students stated that they would appreciate the introduction of an optional module that would educate them on disaster medicine and 94.1% indicated that a knowledge of disaster medicine would be helpful in their future career [3]. Although pharmacists can make many contributions in a variety of emergencies and during a disaster response, their specific role is not well defined [4,5]. Indeed, chemical, biological, radiological, and nuclear (CBRN) risks, including nerve agents, vesicants, radionucleotides, anthrax, and ricin poisoning, could require hospital pharmacies to provide antidotes, antibiotics, antitoxins, and other pharmaceuticals in large amounts [5]. Moreover, with the increasing risk of mass casualty events, pharmacists may also function as first-line responders in case of man-made disasters or as second-line responders caring for victims in health-system or community settings [4]. Therefore, it is crucial to equip pharmacists with the skills and knowledge necessary for any of these eventualities. However, while many pharmacy schools may already be involved with disaster preparedness planning at the local level, there are very few articles on the inclusion of mass casualty education within the undergraduate pharmacy curriculum [6,7].

This study describes the experiences of the Faculty of Pharmacy of Marseille with the development of an elective course on the pharmacist’s role in disaster management and presents an evaluation of the effects of this new teaching module on pharmacy students’ knowledge of disaster response. Students’ self-perceived knowledge, perceptions of teaching quality, and degree of satisfaction were assessed using a voluntary survey.

## Methods

### Ethical statement

According to French law, (Article L1121-1, CSP), ethical approval was not needed for research on teaching practices, including health students. All subjects participated on a voluntary basis.

### Study design

This descriptive study presents the experiences of the Faculty of Pharmacy of Marseille with the development of an elective course on the pharmacist’s role in disaster management, along with an evaluation of the effects of this new teaching module on pharmacy students’ knowledge of disaster response. Students’ self-perceived knowledge, perceptions of teaching quality, and degree of satisfaction were assessed using a voluntary survey.

### Materials and subjects

A new optional 30-hour course on disaster response was implemented in the autumn 2018 semester at the Faculty of Pharmacy of Marseille for third-year pharmacy students. Classes were held on Wednesday afternoons, from 1:30 pm to 5:30 pm, from September 12 to November 15.

### Technical information

A 10-minute overview of this optional course and its content was first presented by an associate professor of the faculty, a military reserve pharmacist, and a pharmacist of the Service de Santé des Armées (SSA) in the format of a PowerPoint presentation to all third-year pharmacy students (220 students) in the teaching amphitheatre, along with the other optional modules (analytical chemistry training for national pharmacy residency examination, pharmacy and communication, introduction to research initiation, introduction to spectroscopy, introduction to the pharmaceutical industry, chemical design of drugs–practical aspects, and biotechnology and health). The syllabus for students was made available online on the Faculty website at https://pharmacie.univ-amu.fr/sites/pharmacie.univ-amu.fr/files/public/2018_2019_3eme_annee_catalogue_ue_a_choix_semestre_5.pdf. The maximum number of accepted students was 60 for each module. The course registration process was conducted through the faculty website.

#### Course format

An expert team of physicians, surgeons and pharmacists of the SSA, pharmacists teaching at the Faculty, and pharmacists from the Bataillon des Marins Pompiers de Marseille (BMPM) was assembled, developed a plan for the program, and scheduled all tasks. Internal and external professionals with expertise in the relevant subject areas were identified. This team of experts established a definition of the goals and objectives and a list of key competencies in line with previous published recommendations [8], a literature review [9], and a subsequent analysis of international guidelines on disaster response training, and identified internal and external professionals with expertise in relevant subject areas who could be invited to give classes to pharmacy students. The objective was not only to improve students’ ability to practice disaster medicine and to prepare them with skills necessary for crisis management. Instead, we considered it necessary for students to understand the pharmacist’s role in crisis management, to be familiar with concepts of disaster preparedness, and to be able to give convincing explanations to calm, comfort, and empower the public.

Facilities for the in-house curriculum units (rooms and equipment) were obtained in coordination with pharmacist school program planners and the lecture support staff.

Lectures were viewed as the best teaching strategy because of the relatively high number of students. Scientific evidence was incorporated and combined with professional expertise to build the content of this course. Because multifaceted stimulating environments stimulate learning and memory [9], the PowerPoint presentations used by each instructor included several illustrations with the goal of provoking as many interactive discussions as possible. As previously described, teaching of problem-solving strategies, presentations of real-life experiences, and role playing were also included to facilitate discussions between the instructor and students [9]. The program and course format were approved by the Formation and University Life Committee of Aix-Marseille University. Course attendance was optional, but students were informed that their knowledge could be assessed through a 5-question multiple-choice quiz at any point after lectures. We decided in advance to conduct 5 evaluations, either at 1:30 pm or 3:30 pm. In addition, the attendance rate was also evaluated at the beginning and the end of each lecture.

#### Students’ learning assessment

As described in a previous study focused on the evaluation of a novel medical student curriculum in disaster medicine [2], students’ knowledge was assessed after each lecture through a multiple-choice quiz written by pharmacists from the SSA. Each question on the 5-question multiple-choice quizzes, phrased as “Which of the following is/are accurate?”, included 5 choices, denoted A, B, C, D, and E (See examples in Appendix 1). One point was given for each question if the response to each statement was correct, 0.5 points if there was 1 error, 0.25 if there were 2 errors, and 0 if there were more than 2 errors. The maximum score was 5. The subject of multiple-choice questionnaires could concern any material covered in a previous session and focused on key competencies defined by the expert team. The testing at course completion also served to fulfil requirements of the third-year pharmacy curriculum. The final minimum score set by the team of experts to denote satisfactory student knowledge was 15 of 25.

#### Students’ satisfaction with the course

The University of Aix-Marseille has a system for students to evaluate teaching. This system is governed by a reference guide and an operational guide of the Evaluation of Teachings by Students, in french Evaluation des Formations et des Enseignements par les Etudiants (EFEE), ratified during the Council of Studies and University Life, in french Conseil des Etudes et de la Vie Universitaire (CEVU) of April 7 and June 5, 2013 and the Board of Directors on June 25, 2013 for questionnaire conceptualization, formatting, and data analysis. Validity was established by a team of experts who responded to the following questions: (1) Is the questionnaire appropriate for the population? (2) Does it represent the content? (3) Is the questionnaire comprehensive enough to collect all the information needed? (4) Does the instrument look like a questionnaire? (5) Is the questionnaire of appropriate length to maximize responses from students?

At the end of the program, on November 15, a voluntary survey was administered to assess students’ self-perceived knowledge, perceptions of teaching quality, and degree of satisfaction. The questionnaire was strictly anonymous and elicited no information revealing students’ identity. The aim of this voluntary survey, the estimated time (10 minutes) required, and a guarantee of anonymity were presented to students. All the information collected was self-reported data. The questionnaire was divided into 2 sections and contained 9 items. The first section comprised questions concerning students’ self-perceived knowledge in crisis management. Section 2 concerned students’ degree of satisfaction with each lecture and global degree of satisfaction (Appendix 2).

### Data availability

For data processing of the survey, the Askabox online survey software was utilized, with pre-developed questions provided by Aix-Marseille University for course evaluations by students. Unfortunately, this website does not retain data for more than 3 months according the recommendations of the French data protection authority (Commission Nationale de l’Informatique et des Libertés, CNIL). Therefore, it is not possible to provide a raw data file; instead, a file with the results of data processing is available (Supplement 1).

## Results

The creation of the final curriculum resulted in a course of 6 modules coupled with 1 visit to a military establishment of medical supplies. The duration of each course was 2 hours (13:30–15:30 and 15:30–17:30), except for the presentation and visit of a military establishment of medical supplies, which comprised 4 hours (Table 1).

The first module (2 hours) focused on the organization and mission of the SSA and introduced the essentials of disaster medicine terminology. Natural and technological disasters, terrorism, man-made disaster, and environmental threats were considered within the framework of the legal environment and regulations for civil protection and disaster preparedness. The functional roles and responsibilities assumed by the SSA and by other authorities involved in crisis management were identified as key competencies.

Module 2 (8 hours) addressed knowledge of CBRN risk management, with descriptions of the recognition and management of incidents involving explosive, chemical, radiological/nuclear, or biological agents, including emergency medical first aid, specific measures, surgical and medical treatment of injuries. Technical equipment, mobile units, protective gear, and contamination detection devices were also presented. The knowledge evaluation was focused on specific dangers of CBRN agents, associated symptoms, self-protection, first-aid medical treatment, and decontamination operations.

The third module (4 hours) discussed the principles of hospital disaster management and preparedness. Mass casualty disposition, the treatment area, transport issues, incident action plans, and information about a functional operations center were presented. As core competencies, notification, deployment, and mobilization actions were taught.

The fourth module (4 hours) taught life-saving and specific emergency medical care for various disaster situations, containing first aid and specific approaches to injuries, burns, explosions, blasts, and illnesses from warfare. Life-saving emergency and limited individual treatment under disaster conditions were discussed. Logistical requirements for the care of burn injuries and high-speed bullet injuries were presented. Presentation of past disasters and reflections on disaster assistance experiences gained from the recent terrorist attacks in Paris and Nice were used to discuss the core competencies.

The fifth module (4 hours) dealt with the pharmacist’s role in drug manufacturing and the military pharmaceutical supply. As key competencies, students were evaluated on their knowledge on factors affecting logistic support in military operations and antidote production.

The sixth module (4 hours) presented the pharmacist’s role from a toxicological perspective. Hazardous chemical accidents, acute poisoning, and toxic syndromes were discussed. This module also dealt with analytical chemistry and toxicology applied to the evaluation of exposure and health risks, as well as practical applications of analytic methods used in toxicology in criminal laboratories. The key competencies for this module were hazard identification, exposure assessment, and management of acute poisoning and intoxication.

The last module consisted of visiting a military establishment of medical supplies and was essentially designed to assess the success of the course.

After the opening of optional course registration, the 60 allotted places in the course were filled in 2 hours, demonstrating significant interest in this course. The average attendance rate at the beginning and the end of each lecture was 97.8%.

### Students’ learning assessment

Concerning students’ knowledge tested through multiple-choice quizzes, the mean and median scores were 19 of 25 and 19.25 of 25, respectively. Two students obtained a final grade of <15 of 25, which in 1 case was due to the student’s absence from an evaluation, 36 obtained a final grade ranging from 15 to 20 of 25, and 22 obtained a final grade of >20 of 25 (Table 2).

### Students’ satisfaction with the course

In total, 59 students (98.3%, 43 women and 17 men) responded to the survey. For 98.3% of them, this elective course allowed them to improve their knowledge in the field of disaster management, and for 70.1% of them, the course was in line with their expectations. This course was not in line with the expectations of 5.2% of the students because they would have appreciated more simulation exercises; furthermore, 5.2% of them considered the course to be “too military,” and the course presented too heavy of a workload for 19.5%. Concerning the teaching quality, the students reported being very satisfied, with a global degree of satisfaction ranging from 3.08 to 3.64 of 4. All students appreciated the modality of 5 multiple-choice quizzes as a way to evaluate their knowledge (Table 3). Finally, 79.3% of students would recommend choosing this optional module next year to second-year pharmacy students.

## Discussion

To the best of our knowledge, few publications have described the development of educational courses for pharmacy students [6,10]. This course was focused on actual scenarios and was in accordance with quality criteria for improving teaching approaches [11]. Although examiners often perceive multiple-choice quizzes as unfair because they may not be sufficient to fully assess the relevance of the course, some studies have demonstrated that multiple-choice quizzes are a well-established and reliable method of assessing knowledge that has a solid relationship with overall competence and performance [12,13]. Moreover, 5 options are commonly used in summative or formative assessments because doing so improves psychometric quality by limiting the effect of random guessing [14]. Of course, this course only enabled students to become familiar with terms and concepts, and did not provide a sufficient framework to retain competencies, which need to be refreshed and assessed through periodic evaluations of knowledge and skills [9]. However, in our experience, this 30-hour course allowed us to provide students with adequate knowledge in a reasonable period and was fully compatible with the educational program and organization of the Faculty of Pharmacy.

The interest expressed by students demonstrated the appeal of this optional module for third-year pharmacy students. With the emerging role of pharmacists in bioterrorism preparedness and disaster response, their integration in the health system is becoming crucial [15]. Indeed, pharmacists provide an effective means of distributing medications, counselling patients, and assisting other members of the health care system. As pharmacists are becoming more integral in the field of CBRN risk, they must efficiently manage these risks and provide information to mass public or information campaigns, because new media are likely to provide flawed information [15].

This voluntary course resulted in satisfactory knowledge of disaster preparedness among the students, with mean scores of approximately 80% on the multiple-choice quizzes, consistent with a previous study [2].

Students’ satisfaction with the course was extremely positive, confirming that most of them (as was found for medical students) would welcome the introduction of an emergency preparedness module into their curriculum [3].

Some limitations of our teaching methods can be noted. First, some students argued that simulation exercises and formal training were lacking in this course. However, some disadvantages of simulation-based medical learning have been identified; for instance, participants will always approach a simulation differently from real life, and only limited amounts of high-quality evidence on the effect and validity of simulation-based training are available [16]. Nevertheless, we will consider this suggestion, and next year a mass triage simulation will be organized by the BMPM to improve students’ understanding of mass casualty management situations. To give students personal experience of how it feels to work in protective equipment, students will also receive practical training in the use of personal protective equipment [9]. This training session will be conducted in a small-group format (5 students associated with 1 BMPM physician) and volunteers will simulate casualties presenting with various disorders, from superficial injuries to moribund conditions and emotional trauma. Students will conduct triage and assume different functional roles. In addition, to demonstrate students’ performance as practising health professionals, a longitudinal evaluation and individualized continuing education plans would be ideal, but these steps may be beyond the scope of our study.

This course was found to have the potential to increase the number of pharmacists prepared to respond to disasters. It also expanded students’ understanding of the pharmacist’s role and stimulated their interest in emergency preparedness. As pharmacists should play an important role in emergency preparedness and response, the introduction of corresponding courses into the standard pharmacy curriculum would be welcome.

## Figures and Tables

**Table 1.** Module content and educational goals

Title	Objectives
Presentation of Service de Santé des Armées	Organisation and mission of the Service de Santé des Armées
Nuclear and radiological risk	Definition of risk; history of environmental protection; sources of contamination; classification of agents
Biological risk	Definition of risk; history of environmental protection; sources of contamination; classification of agents
Chemical risk	Definition of risk; history of environmental protection; sources of contamination; classification of agents
CBRN risk in practice	Demonstration of detection equipment
Exceptional health situations: role of the emergency service (French “SAMU”)	Medical organization in the event of a medical crisis, typical symptoms of CBRN risk, emergency medicine
Exceptional health situations: role of the pharmacist	Role of the pharmacist in cases of exceptional risk, various planning committees, local and state disaster pharmaceutical distribution plans
War wounded and military surgery	Description of war wounded and their injuries - Principles of forward medicalization; damage control
Health risks and epidemiological surveillance in the army	Surveillance of diseases and conditions in the armed forces ; vaccination
Military pharmaceutical supply	Pharmaceutical and logistical constraints in operation; management of blood, oxygen, emergency kits, reagents, and medical biology equipment by the pharmacist
Drug manufacturing	Production of antidotes
Environmental toxicology	Analytical chemistry and toxicology applied to the evaluation of exposures and health risks
Toxicology and criminological investigations	Practical applications of the toxicological methods of analysis used by the police (drugs, detection of counterfeit drugs, etc.)
Presentation and visit of a military establishment of medical supply	Presentation and visit of a military establishment of medical supply

CBRN risk, chemical, biological, radiological, and nuclear risk.

**Table 2.** Student learning assessments for each lecture

Theme	Max score	Average score	Median score	Average score/max score
Nuclear and radiological risk	6	4.59	4.5	0.77
Biological risk	5	4.04	4	0.81
Chemical risk	4	2.68	2.75	0.67
Exceptional health situation	6	4.55	4.5	0.76
Drug manufacturing	2	1.76	2	0.88
Military pharmaceutical supply	2	1.38	1.5	0.69

**Table 3.** Teaching quality and students’ degree of satisfaction

Theme	Easy comprehension of teaching	Quality of the PowerPoint presentation	Pedagogical quality of the speaker	Global degree of satisfaction
Presentation of Service de Santé des Armées	3.41/4	3.39/4	3.69/4	3.50/4
Nuclear and radiological risk	3.52/4	3.39/4	3.69/4	3.53/4
Biological risk	3.45/4	3.37/4	3.71/4	3.51/4
Chemical risk	3.22/4	3.31/4	3.66/4	3.40/4
Exceptional health situations	3.12/4	3.17/4	3.71/4	3.34/4
Drug manufacturing	2.95/4	3.06/4	3.66/4	3.20/4
Military pharmaceutical Supply	2.78/4	2.76/4	3.68/4	3.08/4
Environmental toxicology	3.18/5	3.19/4	3.73/4	3.37/4
Toxicology and criminological investigations	3.57/4	3.52/4	3.81/4	3.64/4
Health risks and epidemiological surveillance in the army	3.26/4	3.28/4	3.74/4	3.43/4
War wounded and military surgery	3.25/4	3.28/4	3.59/4	3.38/4

## Supplementary materials

Supplement 1. Results of data processing

Supplement 2. Audio recording of the abstract.
