# Supplementary material for: Development and evaluation of an elective course on the pharmacist’s role in disaster management in France
Source: J Educ Eval Health Prof. 2019 Jul 15;16:19. doi: 10.3352/jeehp.2019.16.19 (PMC6721963; doi:10.3352/jeehp.2019.16.19)
Supplement: Supplementary file 1 — Supplement 1. Results of data processing [file jeehp-16-19-suppl.pdf]

Supplement 1. Results of data processing

Suivi des réponses à votre questionnaire

Synthèse

Nom questionnaire	UE Menaces et crises sanitaires : le rôle central du Pharmacien
Créateur	marc.montana@univ-amu.fr
Date création questionnaire	08/11/2018
Nombre de questions	8
Nombre de questions obligatoires	0
Nombre de répondants	59

Résultats de votre questionnaire

1. Comment qualifieriez-vous la charge de travail demandée par le cours ?

Nombre de réponses

Nombre de réponses	55
Pourcentage de réponses	93.2%

Synthèse des réponses (total)

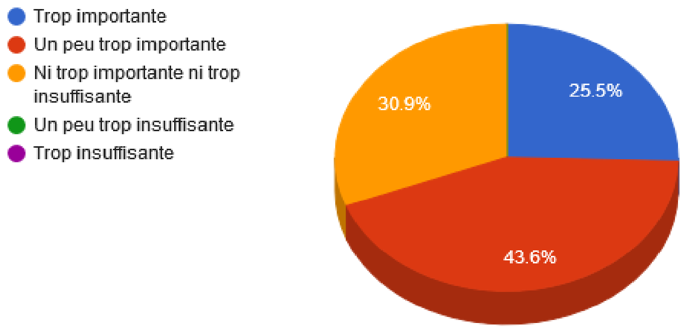

2. Le cours a-t-il répondu à vos attentes ?

Nombre de réponses

Nombre de réponses	57
Pourcentage de réponses	96.6%

Synthèse des réponses (total)

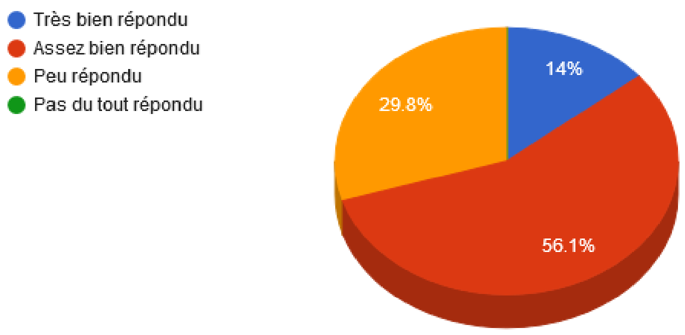

3. Le cours a-t-il contribué à votre connaissance en la matière ?

Nombre de réponses

Nombre de réponses	58
Pourcentage de réponses	98.3%

Synthèse des réponses (total)

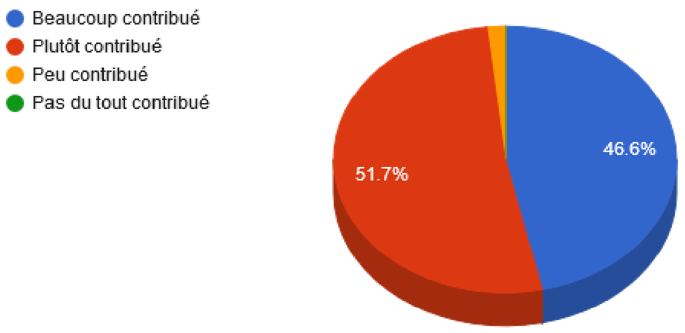

4. Comment qualifieriez-vous le cours ?

Nombre de réponses

Nombre de réponses	57
Pourcentage de réponses	96.6%

Synthèse des réponses (total)

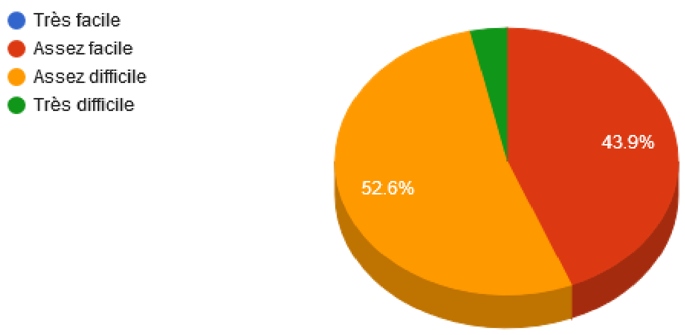

5. Pour chaque cours comment quel est votre degré de satisfaction concernant :
- la qualité des supports de cours utilisés.

Nombre de réponses

Nombre de réponses	58
Pourcentage de réponses	98.3%

Synthèse des réponses (moyenne)

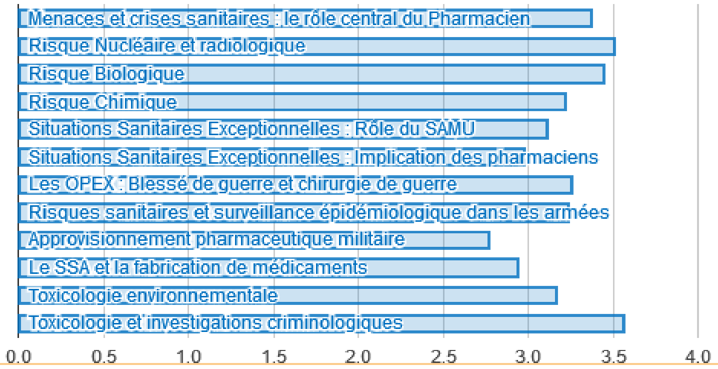

6. Pour chaque cours comment quel est votre degré de satisfaction concernant :
- la clarté du cours

Nombre de réponses

Nombre de réponses	58
Pourcentage de réponses	98.3%

Synthèse des réponses (moyenne)

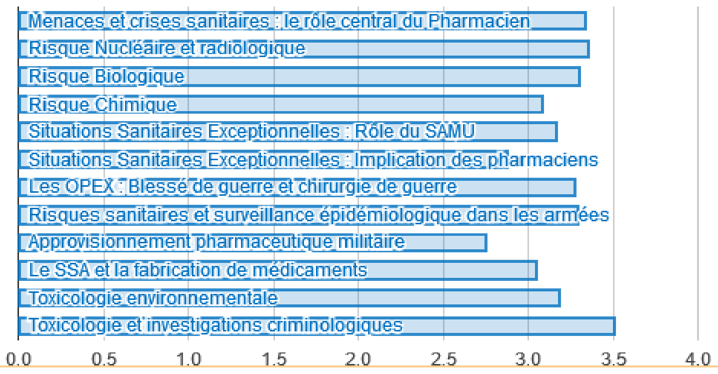

7. Pour chaque cours comment quel est votre degré de satisfaction concernant :
- la disponibilité de l'intervenant.

Nombre de réponses

Nombre de réponses	58
Pourcentage de réponses	98.3%

Synthèse des réponses (moyenne)

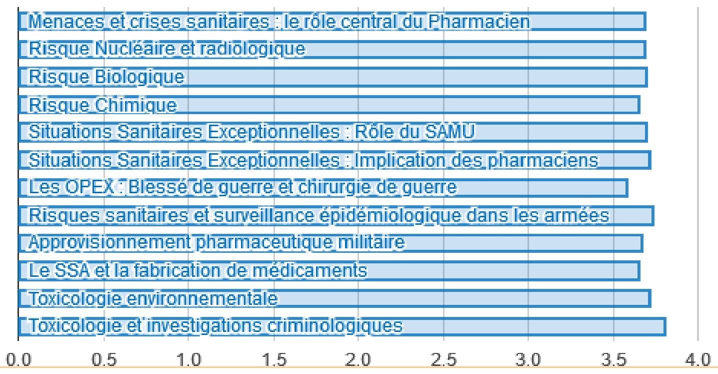

8. Quelle est la probabilité que vous recommandiez le cours à d'autres étudiants ?

Nombre de réponses

Nombre de réponses	58
Pourcentage de réponses	98.3%

Synthèse des réponses (total)

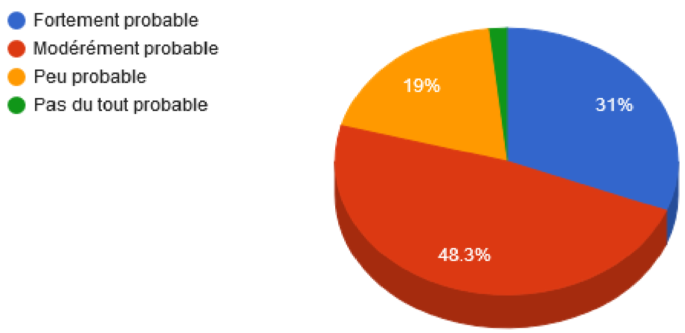

NOM	EVAL 1	EVAL 2	EVAL 3	EVAL 4	EVAL 5	Note /25
**1**	3.5	2.25	4.75	3.75	4.5	18.75
**2**	4.5	3.25	4	5	5	21.75
**3**	4.5	3.5	2.25	5	4	19.25
**4**	4.5	4	3.5	5	3.75	20.75
**5**	5	4	2.5	5	4.5	21
**6**	4	4.5	4.5	4.5	4.25	21.75
**7**	3.25	4	2.25	3.5	4.25	17.25
**8**	3.25	3	2.75	4	4.5	17.5
**9**	5	4.25	3.25	4.5	4.5	21.5
**10**	4.5	2.75	4	5	4.25	20.5
**11**	3.5	0	4.25	4.25	3	15
**12**	3.25	2.75	4	4.25	5	19.25
**13**	2.5	2.25	1.75	2.75	4	13.25
**14**	4.25	4.5	4.5	4.5	4.25	22
**15**	3	2.25	0	3.75	3.5	12.5
**16**	3	3.25	3.25	5	2.5	17
**17**	3.5	2.5	2	3.75	3.25	15
**18**	5	3.5	4	3.5	4.25	20.25
**19**	5	4.5	4.5	5	4.25	23.25
**20**	4	3.5	3.75	4.5	4	19.75
**21**	4.25	3	3.25	5	4	19.5
**22**	4.5	4.25	3.5	3.5	4.5	20.25
**23**	4	4.5	4.5	5	4.5	22.5
**24**	3	2	3.25	4.5	4.5	17.25
**25**	4	3.25	3.25	4.5	3.75	18.75
**26**	5	4.5	4	5	3.5	22
**27**	4	2.75	3.5	4.25	3.5	18
**28**	3.75	4	1.75	5	3.75	18.25
**29**	4.5	3.5	2	5	4.5	19.5
**30**	4.5	3.25	3	4.25	3.75	18.75
**31**	3.25	2.5	2.75	3.25	3.25	15
**32**	3.5	3.25	4.5	2.5	4.5	18.25
**33**	4.5	3.5	3.5	3	3.75	18.25
**34**	3.5	3.25	3	5	4.25	19
**35**	4	4	4.5	3.75	3.75	20
**36**	3.25	2.25	2.75	3.5	3.25	15
**37**	3.5	3.75	2.5	3.75	3.75	17.25
**38**	4.5	4.25	3.25	3	5	20
**39**	4	3.75	4	4	4.5	20.25
**40**	4	4.5	4.5	5	4	22
**41**	4	3	2.25	3.5	4.5	17.25
**42**	4.5	4	2.75	5	4.25	20.5
**43**	3.25	3.5	4	4.25	4	19
**44**	3.75	1.25	4.5	3.25	2.75	15.5
**45**	3.5	3	3.25	3.5	2.5	15.75
**46**	2	2.25	4.5	3.5	3.5	15.75
**47**	4	2.25	2.5	3.75	3.75	16.25
**48**	3	2	2.25	4	4.5	15.75
**49**	4	3	3.25	5	5	20.25
**50**	5	3.25	4	5	5	22.25
**51**	3.75	4.5	4.25	5	3.75	21.25
**52**	4.5	2.5	4	3.25	3	17.25
**53**	4.25	4	3.5	2.5	4	18.25
**54**	4	3.75	4.25	4	5	21
**55**	4.5	3.75	5	5	4.5	22.75
**56**	4.5	5	5	4	4.5	23
**57**	3.75	4	3.5	4	3.5	18.75
**58**	4	4	4	3.5	4	19.5
**59**	5	3.25	3.5	5	4.5	21.25
**60**	2.5	4.5	4	5	3.75	19.75
						18.966667
