# Supplementary material for: Development and evaluation of an elective course on the pharmacist’s role in disaster management in France
Source: J Educ Eval Health Prof. 2019 Jul 15;16:19. doi: 10.3352/jeehp.2019.16.19 (PMC6721963; doi:10.3352/jeehp.2019.16.19)
Supplement: Supplementary file 3 [file jeehp-16-19-app1.pdf]

Appendix 1. Students' learning assessment

UE 31-08C: Menaces et crises sanitaires
EVALUATION n°2 A

Parmi les propositions suivantes concernant les toxiques de guerre, la(les)quelle(s) est(sont) exacte(s)?
(A) Les neurotoxiques organophosphorés (NOP) bloquent la libération d'acétylcholine dans la fente synaptique.
(B) Les NOP ont une structure chimique apparentées à celle de de certains insecticides.
(C) La forme conventionnelle de l'atropine (ampoule 1 mg/1 mL) est adaptée au traitement rapide d'une intoxication aux NOP.
(D) Les hypérites sont susceptibles provoquer des immunodépressions.
(E) Il n'existe aucun antidote contre les agents de guerres cyanés.

Parmi les propositions suivantes concernant les toxiques civils, la(les)quelle(s) est(sont) exacte(s)?
(A) La catastrophe de Bhopal (Inde, 1984) est due à la formation d'un nuage toxique.
(B) Lors de la catastrophe de Bhopal, les victimes ont surtout souffert de symptômes cutanés.
(C) Les catastrophes industrielles sont souvent causées par un seul incident.
(D) La catastrophe de l'usine AZF de Toulouse (2001) est due à un produit de l'industrie pétrolière.
(E) Le monoxyde de carbone est un gaz très toxique mais qui se détecte très facilement.

Parmi les propositions suivantes, laquelle ou lesquelles est (sont) exacte(s)?
(A) Une victime contaminée en surface par un agent chimique (peau, vêtements) peut difficilement contaminer les autres personnes par simple contact.
(B) Lorsqu'une victime est contaminée, le premier geste à réaliser par les secours est l'administration d'antidotes.
(C) Lors de l'attentat au sarin dans le métro de Tokyo en 1995, la décontamination a été efficace.
(D) Le gant poudreur « terre de foulon » s'utilise sur les plaies.
(E) La décontamination de masse peut utiliser des moyens improvisés comme des lances à incendie à pression adaptée.

Parmi les propositions suivantes, laquelle ou lesquelles est (sont) exacte(s)?
(A) Il existe des traitements bien identifiés et efficaces pour traiter les pathologies provoquées par les agents biologiques de la classe 4.
(B) Bacillus anthracis a été utilisé par les allemands pendant la 1ère guerre mondiale pour contaminer la nourriture des animaux.
(C) Le bioterrorisme consiste en l'utilisation d'un agent biologique militarisé, ayant une bonne stabilité au stockage, avec un vecteur de dispersion.
(D) L'épandage est une technique d'aérosolisation dont l'utilisation apparait très vraisemblable dans le cadre d'un acte malveillant.
(E) La toxination consiste en la dispersion d'un agent biologique par le biais d'un réseau d'eau.

Parmi les propositions suivantes, laquelle ou lesquelles est (sont) exacte(s)?
(A) La contamination peut être interne et externe
(B) Le mécanisme d'action biologique porte sur l'ADN
(C) La gravité des effets déterministes ne dépend pas de la dose reçue
(D) Un exemple d'effet déterministe est le cancer radio-induit
(E) Les effets stochastiques sont précoces

UE 31-08C: Threats and health crisis
EVALUATION n°2 A

Which of the following statement(s) about the toxins used in war is/are accurate?
(A) Organophosphorus neurotoxins (NOPs) block the release of acetylcholine into the synaptic cleft.
(B) NOPs have a chemical structure related to that of insecticides.
(C) The conventional form of atropine (1 mg/1 mL ampoule) is suitable for the rapid treatment of NOP poisoning.
(D) Mustard gas is likely to cause immunodepression.
(E) There is no antidote against cyanide.

Which of the following statement(s) about toxicants in civil settings is/are accurate?
(A) The Bhopal disaster (India, 1984) was due to the formation of a toxic cloud.
(B) During the Bhopal disaster, the victims suffered mainly from cutaneous symptoms.
(C) Industrial disasters are often caused by a single incident.
(D) The disaster of the AZF factory in Toulouse (2001) involved a product used in the oil industry.
(E) Carbon monoxide is a very toxic gas, but can be detected very easily.

Which of the following statement(s) is/are accurate?
(A) A victim with superficial contamination by a chemical agent (skin, clothing) is unlikely to contaminate other people by simple contact.
(B) When a victim is contaminated, the first action to be performed by the treatment team is the administration of antidotes.
(C) During the sarin bombing of the Tokyo subway in 1995, the decontamination was effective.
(D) "Fuller's earth" absorbent powder is used on wounds.
(E) For mass decontamination, improvised techniques can be used, such as fire hoses with appropriate pressure.

Which of the following statement(s) is/are accurate?
(A) There are well-identified and effective treatments for the treatment of pathologies caused by class 4 biological agents.
(B) Bacillus anthracis was used by the Germans during the 1st World War to contaminate animal feed.
(C) Bioterrorism consists of the use of a militarized biological agent, with good storage stability, through a dispersion vector.
(D) Spreading of a contaminant is an aerosolization technique whose use appears very likely in the context of a malicious act.
(E) Toxination consists of the dispersion of a biological agent through a water network.

Which of the following statement(s) is/are accurate?
(A) Contamination can be internal and external
(B) The biological mechanism of action of contaminants involves DNA.
(C) The severity of the deterministic effects does not depend on the dose received.
(D) An example of a deterministic effect is radiation-induced cancer.
(E) Stochastic effects occur early.
