# Supplementary material for: Development and evaluation of an elective course on the pharmacist’s role in disaster management in France
Source: J Educ Eval Health Prof. 2019 Jul 15;16:19. doi: 10.3352/jeehp.2019.16.19 (PMC6721963; doi:10.3352/jeehp.2019.16.19)
Supplement: Supplementary file 4 [file jeehp-16-19-app2.pdf]

Appendix 2. Students' satisfaction with the course

QUESTIONNAIRE EVALUATION
UE Menaces et crises sanitaires: le rôle central du Pharmacien

1. Comment qualifieriez-vous la charge de travail demandée par le cours?
◯ Trop importante
◯ Un peu trop importante
◯ Ni trop importante ni trop insuffisante
◯ Un peu trop insuffisante
◯ Trop insuffisante

2. Le cours a-t-il répondu à vos attentes?
◯ Très bien répondu
◯ Assez bien répondu
◯ Peu répondu
◯ Pas du tout répondu

3. Le cours a-t-il contribué à votre connaissance en la matière?
◯ Beaucoup contribué
◯ Plutôt contribué
◯ Peu contribué
◯ Pas du tout contribué

4. Comment qualifieriez-vous le cours ?
◯ Très facile
◯ Assez facile
◯ Assez difficile
◯ Très difficile

5. Pour chaque cours comment quel est votre degré de satisfaction concernant: la qualité des supports de cours utilisés.

	Pas satisfaisant	Peu satisfaisant	Satisfaisant	Très satisfaisant
Menaces et crises sanitaires: le rôle central du Pharmacien	o	o	o	o
Risque Nucléaire et radiologique	o	o	o	o
Risque Biologique	o	o	o	o
Risque Chimique	o	o	o	o
Situations Sanitaires Exceptionnelles: Rôle du SAMU	o	o	o	o
Situations Sanitaires Exceptionnelles: Implication des pharmaciens	o	o	o	o
Les OPEX: Blessé de guerre et chirurgie de guerre	o	o	o	o
Risques sanitaires et surveillance épidémiologique dans les armées	o	o	o	o
Approvisionnement pharmaceutique militaire	o	o	o	o
Le SSA et la fabrication de médicaments	o	o	o	o
Toxicologie environnementale	o	o	o	o
Toxicologie et investigations criminologiques	o	o	o	o

6. Pour chaque cours comment quel est votre degré de satisfaction concernant: la clarté du cours.

	Pas satisfaisant	Peu satisfaisant	Satisfaisant	Très satisfaisant
Menaces et crises sanitaires: le rôle central du Pharmacien	o	o	o	o
Risque Nucléaire et radiologique	o	o	o	o
Risque Biologique	o	o	o	o
Risque Chimique	o	o	o	o
Situations Sanitaires Exceptionnelles: Rôle du SAMU	o	o	o	o
Situations Sanitaires Exceptionnelles: Implication des pharmaciens	o	o	o	o
Les OPEX: Blessé de guerre et chirurgie de guerre	o	o	o	o
Risques sanitaires et surveillance épidémiologique dans les armées	o	o	o	o
Approvisionnement pharmaceutique militaire	o	o	o	o
Le SSA et la fabrication de médicaments	o	o	o	o
Toxicologie environnementale	o	o	o	o
Toxicologie et investigations criminologiques	o	o	o	o

7. Pour chaque cours comment quel est votre degré de satisfaction concernant: la disponibilité de l'intervenant.

	Pas satisfaisant	Peu satisfaisant	Satisfaisant	Très satisfaisant
Menaces et crises sanitaires: le rôle central du Pharmacien	o	o	o	o
Risque Nucléaire et radiologique	o	o	o	o
Risque Biologique	o	o	o	o
Risque Chimique	o	o	o	o
Situations Sanitaires Exceptionnelles: Rôle du SAMU	o	o	o	o
Situations Sanitaires Exceptionnelles: Implication des pharmaciens	o	o	o	o
Les OPEX: Blessé de guerre et chirurgie de guerre	o	o	o	o
Risques sanitaires et surveillance épidémiologique dans les armées	o	o	o	o
Approvisionnement pharmaceutique militaire	o	o	o	o
Le SSA et la fabrication de médicaments	o	o	o	o
Toxicologie environnementale	o	o	o	o
Toxicologie et investigations criminologiques	o	o	o	o

8. Quelle est la probabilité que vous recommandiez le cours à d'autres étudiants?

◯ Fortement probable
◯ Modérément probable
◯ Peu probable
◯ Pas du tout probable

9. Si vous avez d'autres suggestions ou commentaires concernant le cours, merci de nous en faire part.

QUESTIONNAIRE EVALUATION
UE Threats and health crisis

1. How would you describe the workload required by the course?
◯ Too heavy
◯ A little too heavy
◯ Neither too heavy nor too light
◯ A little too light
◯ Too light

2. Did the course meet your expectations?
◯ Very much
◯ Somewhat
◯ Not particularly
◯ Not at all

3. Has the course contributed to your knowledge of the subject?
◯ Very much
◯ Somewhat
◯ Not particularly
◯ Not at all

4. How would you describe the course?
◯ Very easy
◯ Easy
◯ Quite difficult
◯ Very difficult

5. For each session, what is your degree of satisfaction regarding: The quality of the course material.

	Not satisfactory	Unsatisfactory	Satisfactory	Very satisfactory
Presentation of Service de Santé des Armées	o	o	o	o
Nuclear and radiological risk	o	o	o	o
Biological risk	o	o	o	o
Chemical risk	o	o	o	o
Exceptional health situations: role of the emergency service (French "SAMU")	o	o	o	o
Exceptional health situations: role of the pharmacist	o	o	o	o
War wounded and military surgery	o	o	o	o
Health risks and epidemiological surveillance in the army	o	o	o	o
Military pharmaceutical supply	o	o	o	o
Drug manufacturing	o	o	o	o
Environmental toxicology	o	o	o	o
Toxicology and criminological investigations	o	o	o	o

6. For each session, what is your degree of satisfaction regarding: the clarity of the course.

	Not satisfactory	Unsatisfactory	Satisfactory	Very satisfactory
Presentation of Service de Santé des Armées	o	o	o	o
Nuclear and radiological risk	o	o	o	o
Biological risk	o	o	o	o
Chemical risk	o	o	o	o
Exceptional health situations: role of the emergency service (French "SAMU")	o	o	o	o
Exceptional health situations: role of the pharmacist	o	o	o	o
War wounded and military surgery	o	o	o	o
Health risks and epidemiological surveillance in the army	o	o	o	o
Military pharmaceutical supply	o	o	o	o
Drug manufacturing	o	o	o	o
Environmental toxicology	o	o	o	o
Toxicology and criminological investigations	o	o	o	o

7. For each session, what is your degree of satisfaction regarding: the availability of the speaker.

	Not satisfactory	Unsatisfactory	Satisfactory	Very satisfactory
Presentation of Service de Santé des Armées	o	o	o	o
Nuclear and radiological risk	o	o	o	o
Biological risk	o	o	o	o
Chemical risk	o	o	o	o
Exceptional health situations: role of the emergency service (French "SAMU")	o	o	o	o
Exceptional health situations: role of the pharmacist	o	o	o	o
War wounded and military surgery	o	o	o	o
Health risks and epidemiological surveillance in the army	o	o	o	o
Military pharmaceutical supply	o	o	o	o
Drug manufacturing	o	o	o	o
Environmental toxicology	o	o	o	o
Toxicology and criminological investigations	o	o	o	o

8. How likely are you to recommend the course to other students?

◯ Highly likely
◯ Moderately likely
◯ Unlikely
◯ Not at all likely

9. If you have any other comments or suggestions, please let us know.
